# Diagnostic capabilities of self-reported psychoactive substance use among patients admitted to psychiatric consultations in Benin, West Africa

**DOI:** 10.1186/s12888-022-04394-0

**Published:** 2022-11-30

**Authors:** Ahmat K. Abdelhakim, Assad Bio-Sya, Georgia Barikissou Damien, Elvyre T. E. Klikpo, G. M. Gansou, Aurel C. Allabi

**Affiliations:** 1 National Laboratory of Narcotic and Toxicology (LNST), Benin Center of Scientific Research and Innovation (CBRSI), Cotonou, Benin; 2 Faculty of Health Sciences, Laboratory of Pharmacology and Toxicology, University of Abomey-Calavi, Cotonou, Benin; 3 Population and Health Department, Center for Training and Research in Population, University of Abomey-Calavi, Cotonou, Benin; 4 National Center of Psychiatry, Faculty of Health Sciences, University of Abomey-Calavi, Cotonou, Benin; 5 National Laboratory of Narcotic and Toxicology (LNST), National Center of Scientific Research and Innovation (CBRSI), Cotonou, Benin

**Keywords:** Concordance, ASSIST, Self-report, Urine test, Psychoactive substance use, Benin

## Abstract

**Background:**

There is a high prevalence of psychoactive substance use among patients with mental health disorders. The optimal treatment of patients with mental health disorders requires an awareness of their history pertaining substance use. Several methods are used to assess the use of substance. Each of them embodies its limitations. This study aimed at assessing the diagnostic capability of a self-report psychoactive substance use among patients at the National Psychiatric University Hospital of Cotonou, Benin.

**Methods:**

A cross-sectional survey was conducted from August 1, 2021 to November 24, 2021. A total of 157 consenting patients admitted to psychiatric consultations were successively enrolled in the ongoing study. They were screened for the use of psychoactive substance with Alcohol, Smoking and Substance Involvement Screening Test (ASSIST), followed by urine test using the NarcoCheck® kit for qualitative detection of substances or its metabolites. To assess the diagnostic capability, the participants’ self-responses were compared with their urine test results. The sensitivity, specificity, positive and negative predictive values, and kappa coefficient were also calculated.

**Results:**

The frequency of lifetime psychoactive substance use according to self-report was 81.5% (95% CI: 0.746–0.873), while over the past three months (recent use) was 52.2% (95% CI: 0.441–0.603) and 58.6% based on the urine test. Alcohol, tobacco and cannabis were the most prevalent psychoactive substance used. The overall concordance between self-reported psychoactive substance use and the urine test (gold standard) was moderate (sensitivity = 66%; kappa = 0.46). Self-report cocaine use compared with urine test showed the highest concordance (sensitivity = 100%; kappa = 79%), followed by tobacco (sensitivity = 58%, kappa = 41%). On an average 70% of urine test results were consistent with self-report (VPP). Participants’ were more accurate when they were reporting no psychoactive substance use as suggested by the high negative predictive value (NPV).

**Conclusion:**

Diagnostic capability of self-reporting of psychoactive substance use among patients admitted to psychiatric consultations was moderate. Therefore self-reporting may not estimate the exact prevalence of psychoactive substance use. Optimal identification of psychoactive substances use in psychiatric patients requires both history and urine testing. The integration of these two approaches is an excellent method to find out the level, frequency and nature of drug used.

## Introduction

Substance use is becoming more and more rampant worldwide and particularly in some African countries [1–3]. Thereafter, substance use is frequent comorbidity among psychiatric patients [4]. The high prevalence rate of substance use among people with mental health disorders has been well documented in clinical and epidemiological studies. These findings show high risks of substance use among them [5–7]. Substance use disorders (SUD) contribute to 11.8 million deaths globally per year and 1.5% of the global disease burden. It is estimated that 2% of the world population has a SUD, with some countries reporting a prevalence of SUD greater than 5% [8]. In the USA, substance-related disorders affect about 6.1% of Americans, of whom 5.3% have psychiatric disorders [6]. Comorbidity between mental health disorders and substance use disorders (SUD) have been found to be associated with poor treatment outcomes and show a higher psychopathological severity compared to people with a single disorder [9]. It is incumbent upon clinicians to screen substance use early and intervene to mitigate acute risks in a bid to improve the life style trajectory of addiction and its harms [10].

Against this backdrop, screening for substance use is pivotal and crucial to improve the management of patients attending psychiatric care. Numerous methods of screening for substance use have been developed for this purpose [11, 12]. Mainly among these, current measures almost premised on self-report of substance use [13]. A growing body of literature suggests the findings of this method can help to determine the frequency and patterns of substance use, and to assess substance treatment outcomes [10, 11, 14–17]. Self-report is the best performed method using a validated instrument, such as the ASSIST tool [18] that allows for brief but extensive screening for various substances [10]. While self-report is easy, more practical, and less intrusive than laboratory testing, the accuracy of self-report for substance use has been questioned due to real or perceived consequences, the desire to portray oneself positively, and/or respondents’ poor memory of events [16]. With the advent of rapid detection urine toxicology tests, clinicians have new alternatives for diagnosis and patient follow-up. From our knowledge, no studies emerged in West Africa investigating the concordance between self-reporting and urine testing in psychiatric patients. The ongoing study aimed to assess the diagnostic capability of the self-report psychoactive substance use by using the ASSIST questionnaire among patients at the National Psychiatric University Hospital of Cotonou, in Benin.

## Methods

### Study design and participants

A cross-sectional survey was conducted at the National Psychiatric University Hospital of Cotonou, Benin from August 1, 2021 to November 24, 2021. All patients admitted for consultation or emergency care during the study were approached. Patients who were critically unwell and could not be interviewed at presentation were interviewed after stabilization while hospitalized. They were included as they presented to the service and gave free and informed consent to participate in the study. Consent from the participant’s guardian or caretaker was required if necessary. All patients aged 18 years and more were enrolled to participate in the study. The study was conducted after obtaining an approval from the University of Parakou ethics committee for biomedical research (no 0470, July, 26, 2021).

### Data collection and analysis

Two complementary tools were used in this study: i) The Alcohol, Smoking and Substance Involvement Screening Test (ASSIST V3.0) to screen self-reports of substance use and ii) the multi-drug urine kit for toxicology detection. After informed consent, patients were interviewed by using the ASSIST questionnaire. The ASSIST questionnaire was developed by the World Health Organization (WHO) [19]. The ASSIST questionnaire is designed to screen the following psychoactive substance: tobacco products, alcohol, cannabis, cocaine (and derivatives), amphetamine-type stimulants (ATS), sedatives and sleeping pills (benzodiazepines), hallucinogens, inhalants, opioids, and “other drugs”. It is an eight-item questionnaire designed to be administered by a health worker to a patient [20]. ASSIST collects information from patients about lifetime and over the past three-month substance use. The score for each substance is used to quick off a discussion (brief intervention) with patients about their substance use [20, 21].

Following the ASSIST questionnaire, urine samples were collected from patients and analyzed using multi-drug NarcoCheck test (DOA-M12-15B). The multi-drug NarcoCheck® test (DOA-M12-15B) is a homogeneous enzyme immunoassay technique widely used to identify specific substances in human urine. If the urine does not contain the target substance, two red bands (test band and control band) appear on the strip. If the urine sample contained the target substance, only one control band appears on the strip. This multi-drug test consisted of as many urine strips as there are substances to be detected. Each strip therefore detected a specific drug and gives an independent result of a qualitative type “positive” or “negative”. The cut-off of the different substances used in urine screening is shown in Table 1.

**Table 1:** Drug detection windows and cut-offs for urine

Test	Drug class	Cut-off	Detection Windows
THC	Cannabis, marijuana, hashish…	50 ng/ml	2–3 days for occasional use 5 to 15 days for regular use + more than 4 weeks for chronic use
COT	Cotinine	200 ng/ml	1- 2 days after use
COC	Cocaine and crack	300 ng/ml	1–4 days for occasional use + more than 5 days for chronic use
MOR	Morphine, heroin, codeine etc	300 ng/ml	1 to 2 days for occasional use + more than 72 h for chronic use
TML	Tramadol	100 ng/ml	1–3 days after use
BZD	Benzodiazepines	300 ng/ml	3 days for therapeutic use 4–6 weeks for chronic use
AMP	Amphetamines	1000 ng/ml	1 to 2 days after use
MDMA	Ecstasy	500 ng/ml	1–3 days after use
MET	Methamphetamine	1000 ng/ml	1.5—3 days after use
K2	Synthetic cannabinoids of the “Spice” type	25 ng/ml	1—3 days after use
LSD	LSD	20 ng/ml	1—3 days after use
EtG	Ethylglucoronide (alcohol)	300 ng/ml	1—3 days after use

*Abbreviations: THC* delta-9-tetrahydrocannabinol, *MDMA* 3,4-methylenedioxy-N-methylamphetamine, *LSD* lysergic acid diethylamide

Based on urine screening as the gold standard, the validity of the self-reported substance use was assessed in terms of their sensitivity, specificity, positive predictive value (PPV), and negative predictive value (NPV). The Kappa statistic test was calculated to assess consistency in results between self-reported substance use and urine test. The interpretation of the Kappa coefficient was performed according to Landis and Koch (1977) as follows: Kappa values < 0.00, [0.00–0.20], [0.21–0.40], [0.41–0.60], [0.61–0.80] and [0.81–1.00] correspond to the interpretations of “poor”, “slight”, “fair”, “moderate”, “substantial”, “almost perfect” respectively.

## Results

### Socio-demographic characteristics of the respondents

A total of 157 patients participated in the study. The majority of patients were male (75%) with a sex ratio of 3. Average age of the sample was 35 ± 12 years old with extremes of 18 and 70 years old. The most represented age group was 21 to 30 years (32%). The majority of the participants (68.8%) were single. In terms of educational level, 53 (33.8%) participants had secondary education. Most of the patients 50 (31.8%) were not employed. Details of socio-demographic characteristics are shown in Table 2.

**Table 2:** Socio-demographic characteristics of patients (*n* = 157)

Variables	Frequency (%)
**Gender**	
Male	118 (75.0)
Female	39 (25.0)
**Marital status**	
Married	35 (22.3)
Single	108 (68.8)
Divorced	10 (6.4)
widow/widower	4 (2.5)
**Education level**	
Primary	40 (25.5)
Secondary	53 (33.8)
University	39 (24.8)
None	25 (15.9)
**Occupation**	
Farmer/breeder	4 (2.5)
Civil servant	13 (8.3)
Housewife	3 (1.9)
Trader	20 (12.7)
Student	26 (16.6)
No employment	49 (31.2)
employee	42 (26.8)

### Lifetime and current use of substance

**Fig. 1:**
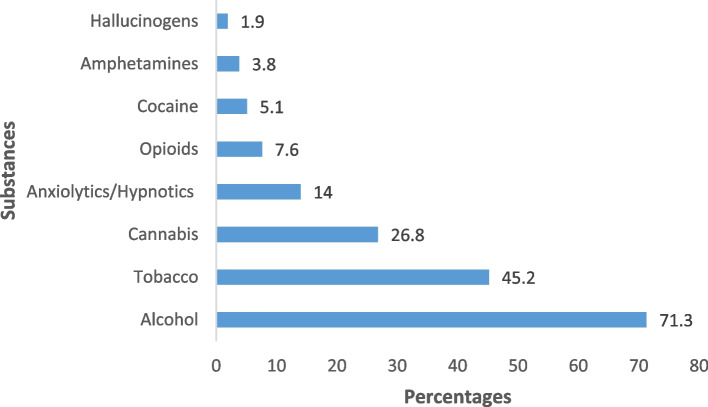
Lifetime frequency of substances use

The lifetime frequency of psychoactive substances use was 81.5%, (95% CI = 74.6% to 87.3%), and it was higher in males (83.9%) than in females (74.35%). Alcohol, tobacco and cannabis were the most commonly reported substances (Fig. 1).

Among the respondents, 38.9% reported using only one substance, and 42.7% reported using two or more substances in their lifetime.

The frequency over the past three months (current use) substance use was 52.2%. Alcohol, tobacco and cannabis were the most commonly reported substances respectively 36.3%, 30% and 11.4%. Details are shown in the Table 3.

**Table 3:** Frequency over the past three months substance use

Psychoactive substances	Never		1–2 times		Monthly		Weekly		Each day or almost	
	n	%	n	%	n	%	n	%	n	%
**Alcohol**	100	63.7	17	10.8	15	09.6	17	10.8	8	5.1
**Tobacco**	110	70.1	12	7.06	5	3.2	6	3.8	24	15.3
**Amphetamines**	157	100	00	00	00	00	00	00	00	00
**Hypnotics/ Anxiolytics**	153	97.5	00	00	2	1.3	00	00	2	1.3
**Cannabis**	139	88.5	6	3.8	3	1.9	9	5.7	0	0.0
**Cocaine**	154	98	00	0.00	1	0.6	1	0.6	1	0.6
**Hallucinogens**	157	100	00	00	00	0.00	0	0.00	0	0.00
**Opioids**	139	88.5	3	1.9	5	3.1	3	1.9	2	1.3

### Urine detection

Table 4 shows the frequency of urine detection of each substance. Overall, the urine test was positive for one or more substances in 58.60% (*n* = 92) of the participants. THC was the most commonly detected substance (35.7%), followed by tobacco (30.6%) and alcohol (21.7%) (Table 4).

**Table 4:** Frequency of urinary detection

Substances	Frequency	Percentage
THC (Cannabis)	56	35.7
Tobacco	48	30.6
EtG (Alcohol)	34	21.7
Cocaine	2	1.3
Morphine	3	1.9
BZD (Anxiolytics/Hypnotics)	22	14.0
Tramadol	8	5.1
LSD	2	1.3

*THC* delta-9-tetrahydrocannabinol, *EtG* Ethylglucoronide, *BZD* Benzodiazepines, *LSD* Diethylamide Lysergic Acid

### Concordance between self-reporting of substance use and urine detection

The sensitivity and specificity of self-reported psychoactive substance use was 66% (0–100%) and 64% (1%-100%) respectively. The positive predictive value (PPV) was 70% (0–75%), and the negative predictive value (NPV) was 60% (69%-100%). When comparing the results of urine test with the results of the self-reported psychoactive substance use within the past three months, we found a kappa value of 0.46, which indicated a moderate concordance between the two tests among different drugs types. This concordance was substantial for cocaine (Kappa = 0.79) and poor for hallucinogens (Kappa = 0.00). The sensitivity, specificity, PPV, NPV and kappa were assessed for each of the psychoactive substance 5 (Table 5).

**Table 5:** Validity of self-reported substance use compared to urine test over the past three months

		**Urine test**				**Urine test**	
**Alcohol**		+	-	**Tobacco**		+	-
**Self-report**	Yes	14	43	**Self-report**	Yes	28	19
	No	20	80		No	20	90
PPV			25%	PPV			60%
NPV			80%	NPV			82%
Se			41%	Se			58%
Sp			65%	Sp			95%
Kappa			0.05	Kappa			0.41
		**Urine test**				**Urine test**	
**Cannabis**		+	-	**Cocaine**		+	-
**Self-report**	Yes	13	5	**Self-report**	Yes	2	1
	No	43	96		No	0	154
PPV			72.2%	PPV			67%
NPV			69%	NPV			100%
Se			23%	Se			100%
Sp			95%	Sp			98%
Kappa			0.21	Kappa			0.79
		**Urine test**				**Urine test**	
**Morphine**		+	-	**Tramadol**		+	-
**Self-report**	Yes	1	4	**Self-report**	Yes	2	3
	No	2	154		No	6	146
PPV			33.3%	PPV			40%
NPV			99%	NPV			96%
Se			33.3%	Se			25%
Sp			97%	Sp			98%
Kappa			0.23	Kappa			0.28
		**Urine test**				**Urine test**	
**Hallucinogens**		+	-	**Anxiolytics/Hypnotics**		+	-
**Self-report**	Yes	0	0	**Self-report**	Yes	3	1
	No	2	155		No	19	134
PPV			0	PPV			75%
NPV			99%	NPV			88%
Se			0	Se			14%
Sp			100%	Sp			1%
Kappa			0	Kappa			0.24
		**Urine test**					
**All substances**		+	-				
**Self-report**	Yes	57	25				
	No	35	40				
PPV			70%				
NPV			60%				
Se			66%				
Sp			64%				
Kappa			0.46				

*PPV* Positive predictive value, *NPV* Negative predictive value, *Se* Sensitivity, *Sp* Specificity

## Discussion

The aim of this study was to assess the diagnostic capability of self-reported psychoactive substance use among patients admitted to psychiatric consultation or emergency department in Benin. To achieve this, the frequency of psychoactive substance use in the lifetime and over the last three months of these patients were determined.

The frequency of self-reported substance use in this study was 81% confirming also the high frequency rate of substance use among people with mental health disorders in our study population. Surprisingly, the frequency reported here is higher than that found elsewhere: 57% in psychiatric emergencies in Philadelphia [22], and 68.5% in Mwanza, Tanzania [23]. The increase in the availability of different substances over time could be one of the major reasons for the exponential growth in substance use.

The pattern of substance use revealed that the most frequently used substance among patients were alcohol (71.3%), tobacco (45.5%) and cannabis (26.8%). Similar profiles of substance use were observed among psychiatric patients in Burkina Faso [4], Tanzania [23] and US [24]. Licit nature of alcohol and tobacco explained their predominance. In the category of illicit substances, cannabis ranked first. The availability and relatively lower cost of cannabis explains its high consumption compared to other illicit substances.

Urine test by NarcoCheck kit was positive in 58.6% of participants, which reflected that the urine test rate was higher than the self-reported substance use (52.2%). The urine test rate found in our study is lower than that reported in a study conducted in the US [22]. This difference could be accounted for by the fact that in our study, a systematic urine test was performed for all participants reported substance use over the past three months, in contrast to the US study which performed the urine test only for participants who reported the use of substance over the past three days. Cannabis (THC) was the most detected substance (35.7%). Several studies have also reported high frequencies of urinary detection of cannabis compared to other drugs [2, 25, 26]. However, the frequency of urine test of cannabis was higher than the frequency of self-reporting use (35.7% *versus* 26.8%). There could be several reasons for the difference between reporting frequency and urine testing. Patients may not want to disclose their use. Moral, socio-cultural and legal restrictions on cannabis use may deter some patients to disclose their use. The stigma associated with use may also reduce the willingness to disclose their use. In addition, the long elimination period of cannabis allows for easy detection, even in subjects whose use is three months old. Contrastively, there was poor detection of alcohol by urine test in this study (21.7 *versus* 36.3 for self-reporting). This is due to the fact that ethylglucuronide is rapidly eliminated from the body, making it difficult to detect in those who have consumed alcohol for more than three days [2, 22]. As a result, urine alcohol tests cannot be used as reference tests. The questionnaire is still of interest in alcohol screening, especially as it provides an opportunity for a nuanced discussion between the patient and the clinician about alcohol consumption. However, the detection of ethylglucuronide in urine can be used as a relapse control marker allowing the clinician to intervene at an early stage [27].

The analysis of the different tests of validity of self-reporting allows to classify the substances into three different categories. In the first category, cocaine and tobacco come first. The sensitivity and specificity observed for these two substances are high, indicating an interesting intrinsic performance for self-reporting using the ASSIST questionnaire.

Cannabis, opioids, anxiolytics and alcohol are in a second category where the sensitivity ranges from 14 to 41%. All of them have a high specificity above 95% except anxiolytics. The kappa coefficient between self-reporting and urine testing for this second category ranges from 0.21–0.40 indicating moderate agreement. Urinalysis for this category adds valuable value to self-reporting. It will help to clarify the diagnosis and to set up a therapeutic follow-up adapted to the incriminated substance.

In a third category occurs alcohol and hallucinogens, substances for which the kappa is close to 0 indicating no agreement. Here, urine tests cannot be used as a reference and other approaches for the detection of those substances should be sought. Against this backdrop, self-reporting provides more information than urine test.

The overall analysis of the results shows that all substances have high negative predictive values. This indicates that patients who declare not to use drugs are telling the truth. Urine test is more recommended for patients who report using at least one substance for further investigation. The positive predictive values found for tobacco, cannabis, cocaine and anxiolytics were all above 60% suggesting good consistency between positive self-report and positive urine test. Similar positive predictive values for tobacco, cannabis, cocaine and anxiolytics have been found in other studies [26, 28, 29]. The extrinsic performance of the self-report, in this case the ASSIST questionnaire, will depend on its intrinsic informative capacity and the context of use.

These different contexts of use explain that several researches found various degrees of concordance between self-reported substance use and urine test [11, 15, 17, 25, 30]. In line with literature, it should be noted that the accuracy of self-reports depends also upon the characteristics and nature of the population involved [11]. In our study, the consistency between self-reported substance use and urine test was moderate. Urine tests are necessary to complete the self-report in order to clarify the diagnosis and to monitor the patient’s treatment. However, in some cases, self-reporting provides more valuable information than urinalysis indicating that the preeminence of each measure will depend on the scenario and the substance. The development of flowcharts integrating different measurement tests ranging from self-reporting to different bioassays for different substances and scenarios will be of paramount support to clinicians and toxicologists in making the appropriate decisions.

By way of conclusion, this study exhibits a moderate diagnostic capability of self-reported psychoactive substance use among patients admitted to psychiatric consultations or emergencies in Benin, suggesting that self-report may not estimate the exact prevalence of substance use. Optimal identification of psychoactive substances use in psychiatric patients requires both history and urine testing. The integration of these two approaches is an excellent method to find out the level, frequency and nature of drug used. A careful integrated interpretation of the two measures is therefore required in psychiatric patients and in general population investigation.

## Data Availability

The datasets used and/or analyzed during the current study are available from the corresponding author on reasonable request.
